# Genome-wide analysis of the TCP gene family and their expression pattern in *Cymbidium goeringii*

**DOI:** 10.3389/fpls.2022.1068969

**Published:** 2022-12-08

**Authors:** Ding-Kun Liu, Cuili Zhang, Xuewei Zhao, Shijie Ke, Yuanyuan Li, Diyang Zhang, Qinyao Zheng, Ming-He Li, Siren Lan, Zhong-Jian Liu

**Affiliations:** ^1^ Fujian Colleges and Universities Engineering Research Institute of Conservation and Utilization of Natural Bioresources, College of Forestry, Fujian Agriculture and Forestry University, Fuzhou, China; ^2^ Key Laboratory of Orchid Conservation and Utilization of National Forestry and Grassland Administration at College of Landscape Architecture and Art, Fujian Agriculture and Forestry University, Fuzhou, China

**Keywords:** genome-wide analysis, TCP gene family, evolution, expression pattern, *Cymbidium goeringii*

## Abstract

TCP gene family are specific transcription factors for plant, and considered to play an important role in development and growth. However, few related studies investigated the TCP gene trait and how it plays a role in growth and development of Orchidaceae. In this study, we obtained 14 TCP genes (*CgTCPs*) from the Spring Orchid *Cymbidium goeringii* genome. The classification results showed that 14 *CgTCPs* were mainly divided into two clades as follows: four PCF genes (Class I), nine CIN genes and one CYC gene (Class II). The sequence analysis showed that the TCP proteins of *C. goeringii* contain four conserved regions (basic Helix-Loop-Helix) in the TCP domain. The exon−intron structure varied in the clade according to a comparative investigation of the gene structure, and some genes had no introns. There are fewer *CgTCP* homologous gene pairs compared with *Dendrobium catenatum* and *Phalaenopsis equestris*, suggesting that the TCP genes in *C. goeringii* suffered more loss events. The majority of the *cis*-elements revealed to be enriched in the function of light responsiveness, followed by MeJA and ABA responsiveness, demonstrating their functions in regulating by light and phytohormones. The collinearity study revealed that the TCPs in *D. catenatum*, *P. equestris* and *C. goeringii* almost 1:1. The transcriptomic data and real-time reverse transcription-quantitative PCR (RT−qPCR) expression profiles showed that the flower-specific expression of the TCP class II genes (*CgCIN2*, *CgCIN5* and *CgCIN6*) may be related to the regulation of florescence. Altogether, this study provides a comprehensive analysis uncovering the underlying function of TCP genes in Orchidaceae.

## Introduction

TCP transcription factors (TFs) compose a gene family particular to plant which relate to growth and development. The gene family was discovered by Cubas et al. (1999) and named according to the TEOSINTE BRANCHED 1 (TB1) gene (*Zea mays*), CYCLOIDEA (CYC) gene (*Antirrhinum majus*), and Proliferating Cell Factor gene (PCF) (*Oryza sativus*) (TCP). TCP genes are characterized by a basic helix-loop-helix motif of 59 amino acids (aa) named the TCP domain, which allows protein interaction and DNA binding (Kosugi & Ohashi, 1997; Cubas et al., 1999; Shang et al., 2022). TCP genes are mainly divided into Class I and Class II according to the loss of four amino acids in Class II basic domains (Martín-Trillo and Cubas, 2010). Class I, also called the TCP-P class, contains the PCF genes PCF1 and PCF2, which were discovered in rice and bind the promoter of the PROLIFERATING CELL NUCLEAR ANTIGEN (PCNA) gene, which is related to DNA replication, chromosome maintenance and cell cycle progression (Cubas et al., 1999). Class II was also known as the TCP-C class and contains the TB1, CYC, and CIN genes. The TB1 genes mainly regulate apical dominance and inflorescence development (Doebley et al., 1997; Dixon et al., 2018), the CYC genes mainly control floral bilateral symmetry development (Luo et al., 1999; Hileman, 2014), and the CIN genes are mostly involved in the development of lateral organ (Palatnik et al., 2003; Nath et al., 2003; Crawford et al., 2004; Walcher-Chevillet and Kramer, 2016). More non-model plant TCP genes were identified and analyzed in the genome era, promoting the comprehension of the structures and functions of this gene family.

TCP transcription factors are ancient proteins that exist in all plant phylostrata, except for unicellular algae (Navaud et al., 2007; Floyd & Bowman, 2007). This gene family contains only five to six members in moss, ferns, and lycophytes. With genome duplication, evolution and diversification, more than ten genes have been generated in angiosperms and gymnosperms, and more than 20 members have been generated in some model plant species (Riechmann et al., 2000; Cubas, 2002; Navaud et al., 2007; Mao et al., 2014). Two classes of genes exist in all plants that contain TCP genes, but the CYC/TB1 genes were not found in lycophytes or more ancient plants (Floyd & Bowman, 2007; Horn et al., 2015). Therefore, the CIN class is more ancient than CYC/TB1, and the CYC/TB1 clade may have originated in gymnosperms or angiosperms and is mainly involved in floral organ development (Palatnik et al., 2003; Koyama et al., 2007; Horn et al., 2015). The TB1 genes and homologs mainly control leaf axillary development in vegetable and reproductive organs of monocots (Hubbard et al., 2002; Lewis et al., 2008; Yuan et al., 2009; De Paolo et al., 2015). The CYC1/2/3 genes perform different functions in core eudicots (Howarth & Donoghue, 2006). The number and function of TCP genes varied and differentiated during plant evolution.

Orchidaceae is one of the most diverse families in vascular plants, containing about 28,000 species in 700 genera (Chase et al., 2015; Christenhusz & Byng, 2016; Zhang et al., 2017). Orchids grow in an extensive range of habitats worldwide, with high diversity in flower and vegetable morphology, life form, and pollination (Givnish et al., 2015; Chen et al., 2020a; Chen et al., 2021; Liu and Lan, 2022; Li et al., 2022). Although orchids have high biological and economic value, few studies investigating TCP TFs have been performed in orchids, and the floral morphology and reproductive development are still unknown. Lin et al. (2016) identified 23 TCP genes in *Phalaenopsis equestris*, focused on the development of ovules, and discovered that *PePCF10* and *PeCIN8* influence cell division to play crucial roles in ovule formation. Zhang et al. (2021a) identified 25 TCP genes in *Dendrobium catenatum* and concentrated on the TCP genes that control leaf development through the jasmonate-signaling pathway. *DcaTCP4* and *DcaTCP9* were found to possess varied expression patterns after 3 h of jasmonate treatment. Recently, some orchid genomes have been sequenced and analyzed (Sun et al., 2021; Yang et al., 2021; Ai et al., 2021; Li et al., 2022), and a TCP gene family analysis could be promoted.

To demonstrate the properties of TCP genes over the evolution of orchids, we undertook genome-wide identification, comparison, and expression investigations of TCP genes in *Cymbidium goeringii* (Spring Orchid or Chunlan) (Sun et al., 2021) in this work. The findings could offer novel understanding of the fundamental processes driving the growth and development of orchid organs and other flowering plants.

## Materials and methods

### Plant materials

Wild plants of *C. goeringii* were cultivated and collected from the Forest Orchid Garden greenhouse at Fujian Agriculture and Forestry University (Fuzhou, Fujian Province, China). The temperature of the greenhouse culture was approximately 25°C. The roots, stems, leaves and flowers (sepal, petal, lip and column) were used in this study. All samples were gathered, put in tubes, and frozen before being stored in an ultralow temperature refrigerator at −80°C.

### Genome-wide identification and physicochemical properties of TCP genes

*CgTCP* (*C. goeringii* TCP gene) genes were identified in the *C. goeringii* genome (Sun et al., 2021), and five species (*Arabidopsis thaliana*, *Oryza sativa*, *Ananas comosus*, *Phalaenopsis equestris*, and *Dendrobium catenatum*) TCP proteins served as the queries. The genome and transcriptome data of *C. goeringii* were downloaded from the NCBI database^1^, and the *Arabidopsis thaliana* TCP proteins were downloaded from TAIR^2^, the rice TCP proteins download from Rice Genome Annotation Project (http://rice.uga.edu/), the *Ananas comosus*, *Phalaenopsis equestris*, and *Dendrobium catenatum* TCP proteins download from previous studies (Lin et al., 2016; Zhang et al., 2021a; Wang et al., 2022). BLAST and HMMER were used to identify the TCP genes. A BLAST table (E-value < 0.05) and sequence were acquired from TBtools (Chen et al., 2020b). The hidden Markov model (HMM) of the TCP domain (PF03634) was downloaded from the Pfam protein family database^3^, and the HMM profile was used to identify the TCP protein sequences through the Simple HMM Search in TBtools (Chen et al., 2020b). Combining the results of BLAST and Hmmsearch, all TCP genes were analyzed by NCBI Batch-CDD tools^4^, and the genes containing the entire TCP domain were retained. The ExPASy online tool^5^ (Artimo et al., 2012) used to predict physicochemical properties of the TCP genes, and the Plant-mPloc^6^ (Chou and Shen, 2010) used to predict subcellular localizations.

### Motif and gene structure analysis of TCP genes

According to the gene location of the TCP genes in the *C. goeringii* genome, complete gene sequences were extracted. Complete gene sequences and coding sequences (CDSs) were prepared for the gene structure analysis in GSDS^7^ (Hu et al., 2015). The MEME^8^ online tool (Bailey et al., 2009) used the default parameters to examine the motifs of TCP genes, and the visualization of the results of the gene structure and motifs were combined using TBtools.

### Phylogenetic analysis and classification of TCP genes

The TCP genes of *C. goeringii* were collected and identified, and the sequences were aligned by MUSCLE (Edgar, 2004) using MEGA 7 (Kumar et al., 2016). Alignment was also conducted in the TCP genes of *C. goeringii*, *Arabidopsis thaliana*, *Phalaenopsis equestris* (Lin et al., 2016) and *Dendrobium catenatum* (Zhang et al., 2021a). The phylogenetic analysis was performed by the maximum likelihood (ML) method using RA×ML (RAxML-HPC2 on XSEDE) in the CIPRES Science Gateway web ^server9^ (Miller et al., 2010) with the Protein CAT model and GTR matrix with 1,000 bootstrap iterations. The Evolview (He et al., 2016) used to polish the output phylogenetic tree.

### Collinearity analysis and mapping TCP genes on chromosomes

The protein sequences (PEPs), CDSs and annotation files (gff files) of the *C. goeringii*, *D. catenatum* and *P. equestris* genomes were prepared for a collinearity analysis and gene mapping. The TCP gene location information in the *C. goeringii* genome chromosomes was acquired in the gff file, and visualization was conducted by the R package ‘RIdeogram’ (Hao et al., 2020). JCVI v1.2.10 (https://pypi.org/project/jcvi/) was used in the collinearity analysis of *C. goeringii*, *P. equestris* and *D. catenatum*. First, the PEP, CDS and gff files were formatted. Second, the PEP files were aligned and analyzed to acquire the collinearity file. The anchor file was used in the visualization, and the TCP genes were highlighted in the collinearity map.

### Prediction of *Cis*-acting elements

In total, 2000 bp upstream sequences of TCP genes were retrieved to investigate the regulatory functions in plant growth and development. The sequences were extracted by TBtools according to the gene locations in the annotation file. The *cis*-acting elements of the TCP genes were identified and annotated in the promoter regions by the online tool PlantCARE^10^ (Lescot et al., 2002). TBtools (Chen et al., 2020b) was used to display the findings of the *cis*-acting element counts and annotation.

### Expression pattern analysis

RNA-Seq by Expectation Maximization (RSEM) (Li & Dewey, 2011) was employed for the transcript quantification and calculation of the fragments per kilobase per million mapped reads (FPKM) value of each gene in the transcriptomic analysis. The FPKM matrix and heatmaps were generated by TBtools (Chen et al., 2020b).

Roots, stems, leaves, and mature flowers (sepal, petal, lip, and column) from *C. goeringii* grown at the Forest Orchid Garden of Fujian Agriculture and Forestry University were taken for a quantitative real-time PCR (qRT-PCR) analysis to confirm the expression pattern of the TCP genes. Three replicates of each type of tissue were sampled in the analysis. The total RNA was extracted from the tissues by RNA Simple Plant Kit. The RNA concentration in each tissue was in the range of 34.3–398.9 ng/µl, with A260/280 values ranging from 2.01 to 2.14, indicating that the extracted RNA was of high quality. The primer tool in Geneious (Kearse et al., 2012) was employed to design specific PCR primers. In **Supplementary Table 1**, the gene-specific primers for the three candidate genes are given together with the associated internal reference genes. The cDNA synthesis and qPCR were carried out using the Vazyme/R223 and Yeasen/11202ES03 kits, respectively. RT−qPCR was carried out to confirm the precise expression of Class II genes in the roots, stems, leaves and flowers using CIN genes in *C. goeringii* (GL10643, GL16029 and GL28896). Three biological replicates and three technical replicates were used in each experiment. The relative gene expression was calculated using the 2^−ΔΔCT^ method.

### Gene ontology analysis

The protein file of the *C. goeringii genome* was used to search against the eggNOG 5.0 database using EggNOG-mapper v2^11^ (Huerta-Cepas et al., 2019) for Gene Ontology (GO) functional annotation. Sequence similarity was used to predict function, sequence alignment was used to predict orthology, E-values and bit scores were used to filter out low-quality orthology alignments, and GO annotation terms associated with proteins involved in well-known biological processes were used to classify functions.

## Results

### Identification and protein traits of TCP genes

In total, 14 full-length TCP genes were identified from the *C. goeringii* genome. The **Supplementary Data Sheet** contained the complete protein sequences for TCP. InterproScan 5 (Jones et al., 2014) was used to confirm that all potential TCP genes encode the conserved TCP domain. The 14 TCP genes had an average length of 323 aa (range 169–572 aa), which was located on eight chromosomes. The average molecular weight (MW) was 35321.21 kDa (range 19216.05–63038.55 kDa). The grand average of hydrophilic (GRAVY) values were all negative in *C. goeringii* TCP proteins, suggesting genes with strong hydrophilicity. The average isoelectric point (pI) of the TCP proteins was 8.5 (range 6.60–10.55), indicating relatively strong acidity. The results of the subcellular location predictions revealed that all TCP proteins were found in the nucleus, except for one gene (*GL08210*), suggesting that they mostly function as TFs in the nucleus (**Table 1**).

**Table 1:** Analysis of amino acid sequence characteristics of the CgTCP gene family in *Cymbidium goeringii*.

Gene name	Gene ID	Chromosomal location	Number of amino acids	Molecular weight	Theoretical pI	GRAVY	Subcellular localization
CgPCF1	GL09311	Chr13:87180856–87181647	263	28413.69	7.17	-0.551	Nucleus.
CgPCF2	GL11234	Chr04:49748318–49749121	267	28703.48	9.73	-0.351	Nucleus.
CgPCF3	GL13021	Chr03:184402053–184416102	411	45008.03	10.55	-0.535	Nucleus.
CgPCF4	GL20265	Chr03:9200177–9200866	229	23851.91	8.66	-0.321	Nucleus.
CgCIN1	GL08210	Chr09:205279584–205289529	360	40250.07	8.64	-0.502	Chloroplast. Nucleus.
CgCIN2	GL10643	Chr13:137361269–137362399	376	40749.62	7.23	-0.513	Nucleus.
CgCIN3	GL14058	Chr08:24486362–24488656	572	63038.55	6.6	-0.399	Nucleus.
CgCIN4	GL14488	Chr13:164527392–164528508	195	21815.39	9.42	-0.835	Nucleus.
CgCIN5	GL16029	Chr03:237463322–237471452	438	47532.56	7.23	-0.321	Nucleus.
CgCIN6	GL28896	Chr01:265107853–265111194	325	34518.12	6.66	-0.439	Nucleus.
CgCIN7	GL29216	Chr11:158943146–158944616	226	25115.2	9.51	-0.692	Nucleus.
CgCIN8	GL29377	Chr08:193755574–193757553	169	19216.05	9.41	-0.525	Nucleus.
CgCIN9	GL31284	Chr01:275094509–275096742	297	33298.18	9.37	-0.709	Nucleus.
CgCYC1	GL16832	Chr16:45535648–45547591	388	42986.76	8.92	-0.495	Nucleus.

### Phylogenetic analysis of the protein sequence

**Figure 1:**
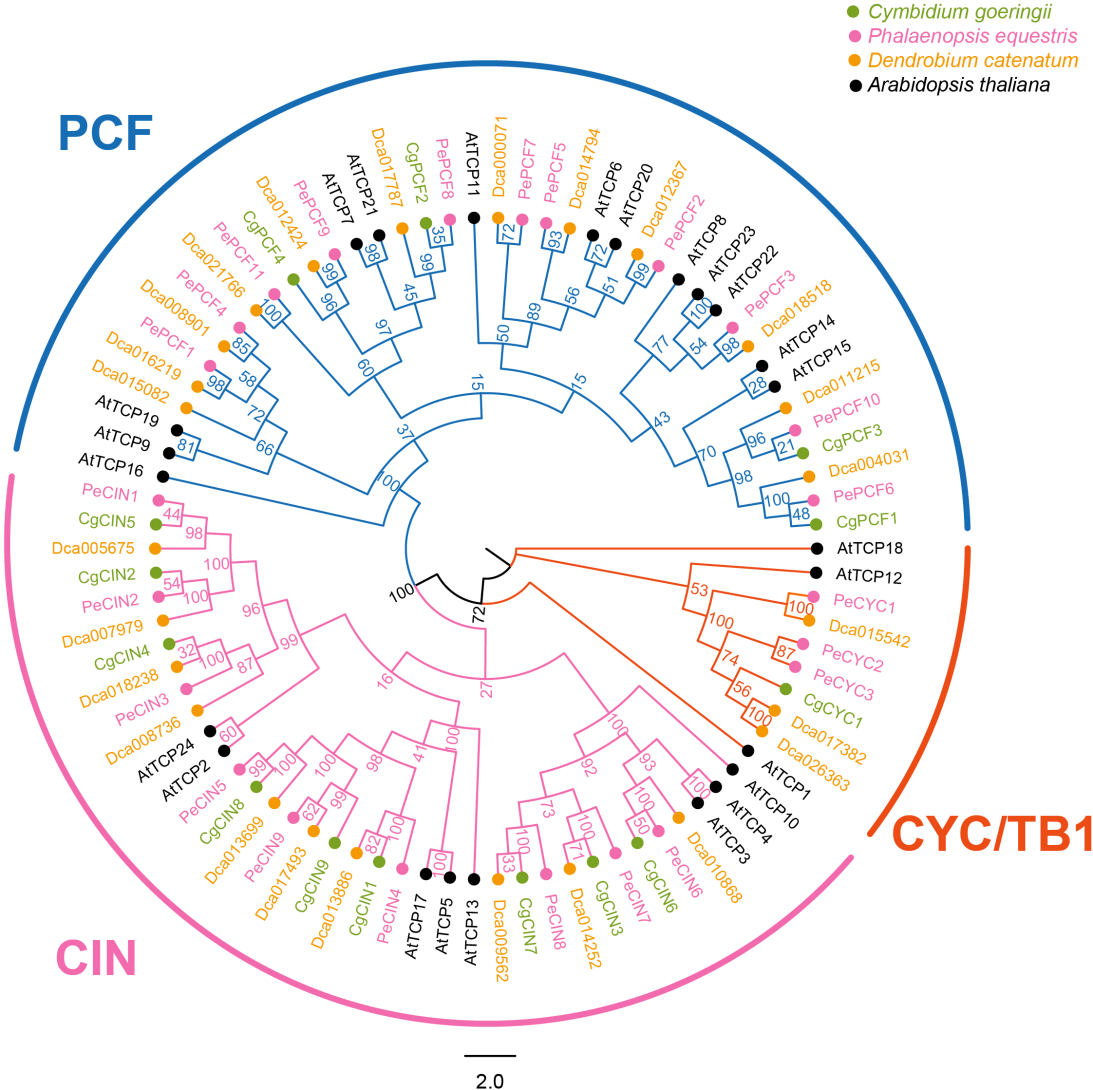
Phylogenetic tree based on the TCP protein sequences of *Cymbidium goeringii*, *Phalaenopsis equestris*, *Dendrobium catenatum* and *Arabidopsis thaliana*. Phylogenetic analysis indicated that the TCP gene family was classified into the following two clades: Class I (PCF) and Class II (CIN and CYC/TB1); there are two subclades in Class II. TCP protein sequences of *C. goeringii* are available in the **Supplementary Data Sheet**.

TCP genes of four species (*Arabidopsis thaliana*, *Phalaenopsis equestris*, *Dendrobium catenatum* and *C. goeringii*) were used to conduct the phylogenetic analysis. In total, 87 genes in four species were classified into two clades (**Figure 1**). In total, 14 genes of *C. goeringii* were divided into two clades, Class I (PCF genes) and Class II (CIN genes and CYC/TB1 genes). Similar to other orchids, three types of genes exist in *C. goeringii* as follows: four PCF genes, nine CIN genes and one CYC gene. We also performed the TCP proteins phylogenetic analysis of 18 different plant phylostrata groups species, results showed Class I and Class II (Class II-A, B, C) were divided (**Supplementary Figure 1** **;** **Supplementary Table 2**). The TCP protein of algae only found in Class I and orchids TCP proteins were scattered in different clades.

**Figure 2:**
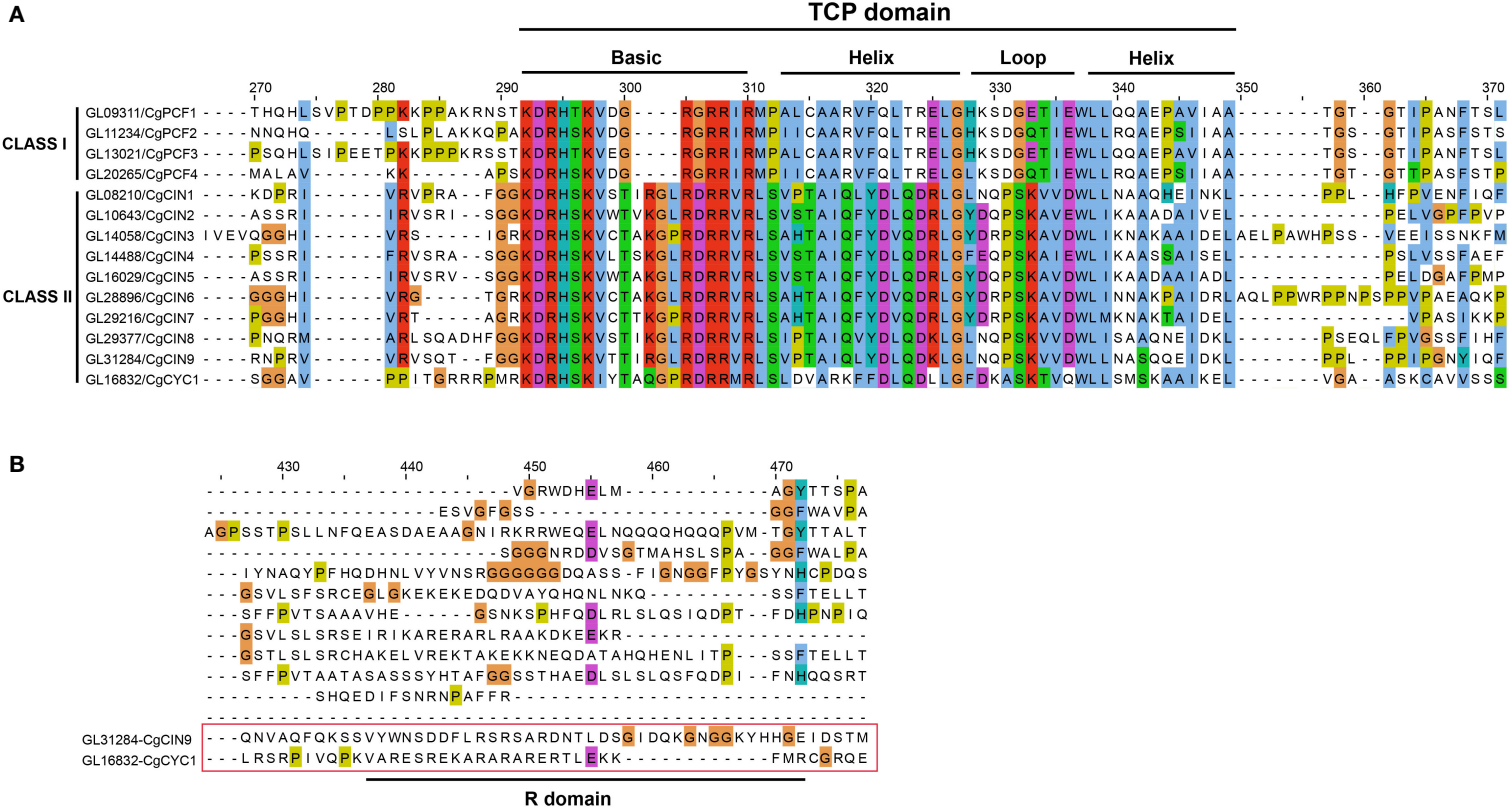
Multiple sequence alignment and protein sequence of the TCP domain. **(A)** Alignment of the TCP domain containing the Basic, Helix and Loop sequences for the predicted *C. goeringii* TCP proteins. **(B)** Alignment of the R-domain of the CIN and CYC genes. .

We also analyzed the sequence alignment of CgTCP genes. The TCP domain was present in all CgTCP genes, and the domain can be divided into four parts (**Figure 2A**, **Supplementary Figure 2**). The basic region was the most conserved, followed by two helix regions, and the loop region was more varied than the other regions. The TCP domain of the CgTCP proteins was similar to that in *P. equestris* and *D. catenatum* (Lin et al., 2016; Zhang et al., 2021a), indicating that the TCP domain is highly conserved in orchids. One CgTCP protein (*CgCYC1*) exited an R domain compared with *CgCIN9* (**Figure 2B**), and the R domain was only present in the CYC gene, which is consistent with the phylogenetic results.

### Collinearity analysis of TCP genes between orchids

**Figure 3:**
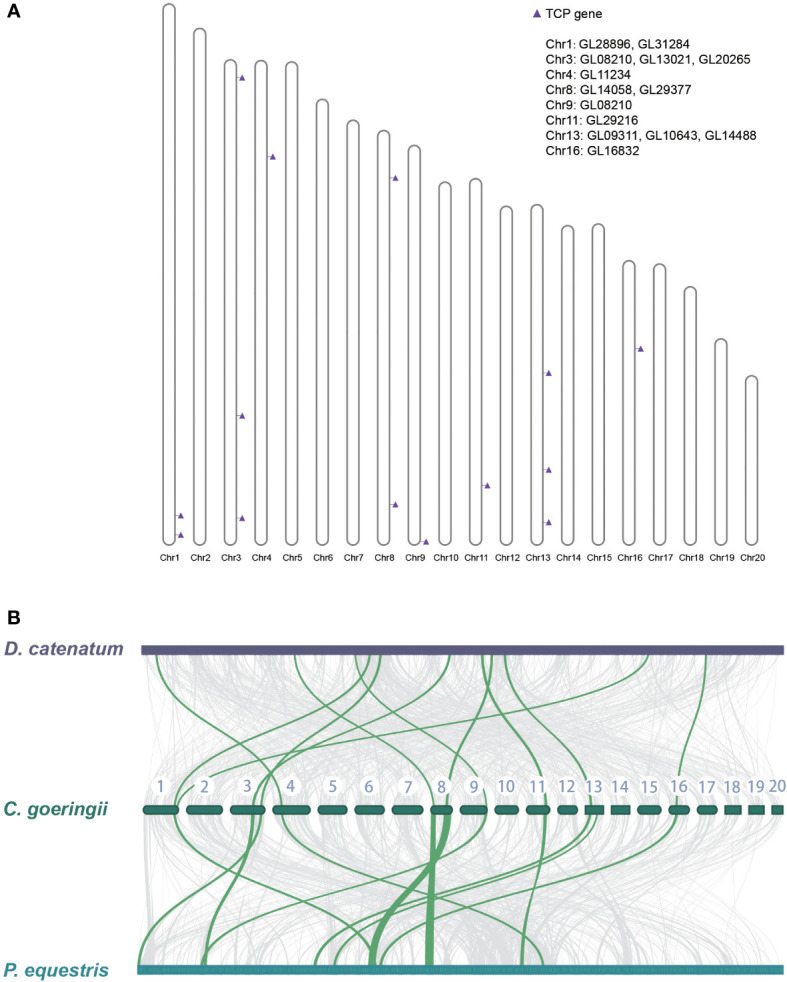
The TCP gene location on the chromosome of *C. goeringii* and collinearity between *P. equestris* and *D*. *catenatum*. **(A)** The chromosomal location of *CgTCP* genes. **(B)** The collinearity analysis suggested that the three orchids presented nearly one-to-one correspondence of TCP proteins.

The gene location on the chromosome of *C. goeringii* was examined, and 14 TCP genes were located on eight chromosomes (**Figure 3A**). In addition, a collinearity analysis was conducted among the TCP genes of *C. goeringii*, *P. equestris*, and *D. catenatum*. The collinear relationship among the TCP genes was examined to identify potential duplication events during TCP gene evolution in orchids. The results demonstrated a nearly 1:1 correspondence between all TCP genes in the three orchids, indicating minimal genomic rearrangements and TCP orthologs reshuffling after the lineages of *Dendrobium* and *Cymbidium* diverged (**Figure 3B**). The results showed that gene pseudogenization and loss might lead to a decrease in TCP in *C. goeringii*.

### Gene structure and motif analysis

**Figure 4:**
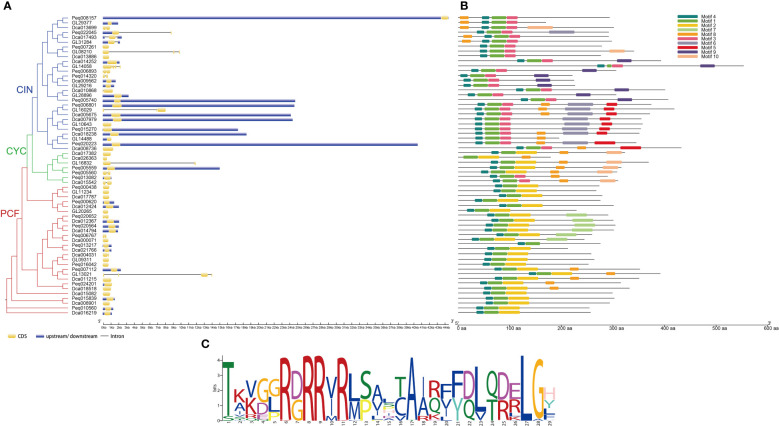
Gene structure, conserved motifs, and conserved domains of TCP genes. **(A)** Phylogenetic tree and gene structure of the TCP genes of *C. goeringii*, *P. equestris* and *D*. *catenatum*. **(B)** Predicted motifs of the TCP genes of *C. goeringii*, *P. equestris* and *D*. *catenatum*. **(C)** Sequence logo of motif 1, which encodes the TCP domain.

To explore the gene structure of TCP genes in orchids, the intron–exon and up/downstream regulatory element distributions of *C. goeringii*, *P. equestris* and *D. catenatum* were analyzed (**Figure 4A**). The results revealed that the TCP family of *C. goeringii* had 1–5 exons and 0–4 introns, *P. equestris* had 1–3 exons and 0–2 introns, *D. catenatum* had 1–2 exons and 0–1 intron. Although similarity in the gene structure was found in each subclade, the TCP genes of *C. goeringii* have a high degree of variance in the intron length and exon numbers in comparison with *A. thaliana*, *P. equestris* and *D. catenatum*. In general, most Class I genes exhibited fewer exons than the Class II genes. Notably, in Class I, *CgPCF1*, *CgPCF2* and *CgPCF4* have no exons, whereas in Class II, *CgCIN2* and *CgCIN4* have no exons (**Figure 4A**).

The motifs of the TCP proteins in *C. goeringii*, *P. equestris* and *D. catenatum* were analyzed by the MEME, and 10 motifs were set as upper bounds (**Figure 4B**). The number of TCP motifs both ranged from three to six in *C. goeringii* and *D. catenatum*, and ranged from two to six in *P. equestris*. Motif 1 encoded the TCP domains (**Figure 4C**). The PCF proteins all contain motif 1, motif 2 and motif 4, CIN protein all contain motif 1 and motif 3, the PCF proteins were more conserved compare with CIN and CYC proteins by motif analysis.

### *Cis*-acting regulatory elements and GO enrichment analysis

**Figure 5:**
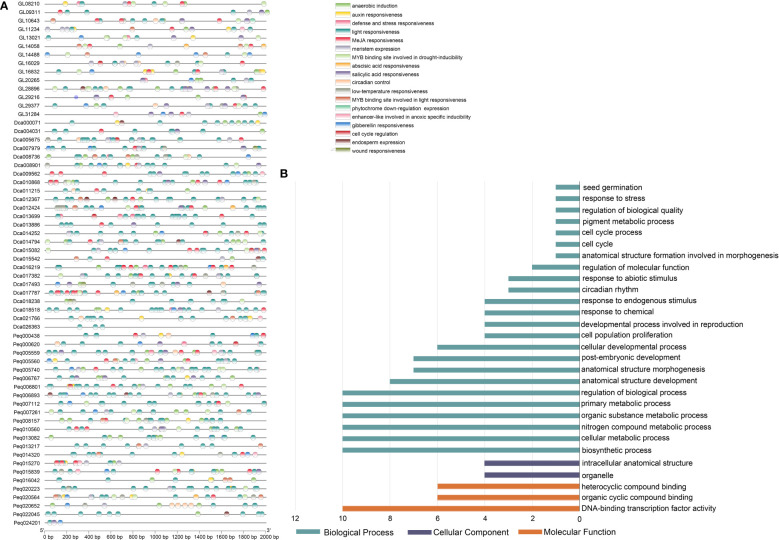
*Cis*-acting elements and GO enrichment analysis in the promoter regions of TCP genes. **(A)** Elements analysis of *C. goeringii*, *P. equestris* and *D*. *catenatum*, the same regulatory functions are presented in the same color. **(B)** Gene Ontology (GO) terms of *C goeringii* TCP genes; the detailed data are listed in **Supplementary Table 5**.

The 2,000 bp upstream regions of *C. goeringii*, *P. equestris* and *D. catenatum* TCP genes were extracted for the identification of putative *cis*-elements and used to explore the promotor region functions. In total, 1644, 2250, 2440 *cis*-acting elements attributed to 26, 26, 29 types and 9, 9, 10 responsive functions were identified in *C. goeringii*, *P. equestris* and *D. catenatum*, respectively (**Figure 5A** and **Supplementary Table 3**). TATA-box made up the majority of these components (67.76%, 52.18%, 46.31%), followed by CAAT-box (11.25%, 12.80%, 15.49%) in *C. goeringii*, *P. equestris* and *D. catenatum*, respectively (**Supplementary Table 4**). The *cis*-element functions included phytohormone responsiveness to auxin, abscisic acid (ABA), gibberellin (GA), methyl jasmonate (MeJA) and salicylic acid; stress responsiveness, such as anoxic, anaerobic, drought and low-temperature stress; and growth and development elements, such as light response, cell cycle regulation, endosperm expression, meristem expression and circadian control (**Figure 5A**). Light responsiveness was the most prevalent element function in each TCP gene, suggesting the crucial functions that light plays in modulating TCP function throughout plant growth and development (**Figure 5A**). The second and third most prevalent types were MeJA-responsive and ABA-responsive elements, which were likewise broadly distributed in the majority of orchid TCPs (**Supplementary Table 5**). These results suggest that these elements may have roles in controlling these two phytohormones. We also discovered other components involved in meristem expression and meristem-specific activation, which is in line with the crucial functions played by TCP in the preservation of meristem homeostasis.

A GO analysis was performed to annotate the gene functional classifications of the TCP genes of *C. goeringii* and investigate the important biological processes (**Figure 5B**). As a result, the GO terms “cellular developmental process”, “intracellular anatomical structure” and “DNA-binding transcription factor activity” were the most related to plant growth and development in the GO ontologies “Biological Process,” “Cellular Component,” and “Molecular Function,”, respectively (**Figure 5B** and **Supplementary Table 6**). The findings are consistent with the fact that TCP is a premier regulator involved with a number of downstream transcriptional networks and functions mostly acts in the nucleus (**Table 1**). Several other terms, such as “circadian rhythm” and “seed germination”, are consistent with TCP’s function.

### Expression pattern analysis of TCP genes

**Figure 6:**
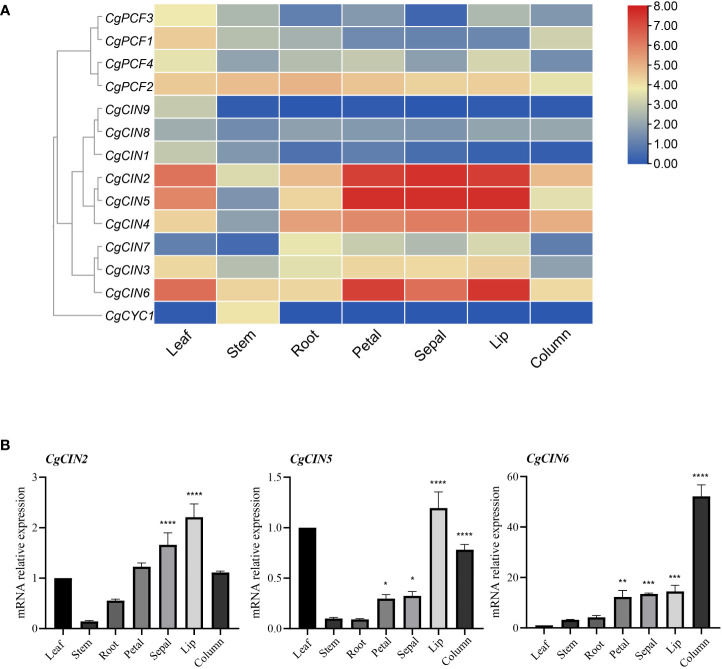
The expression pattern and RT−qPCR verification of TCP genes in seven tissues in *C. goeringii*. **(A)** Expression heatmap of TCP genes in seven tissues in *C. goeringii.* **(B)** Expression pattern of *CgCIN2*, *CgCIN5* and *CgCIN6 in seven tissues* in *C. goeringii* by RT−qPCR. We performed the ANOVA multiple comparisons in the statistical test, star marks *, **, ***, and **** representing adjusted p < 0.05, p < 0.01, p < 0.001, and p < 0.0001, respectively (**Supplementary Table 8**).

Based on transcriptome data from *C. goeringii* tissues, including flowers, leaves, stems, and roots, an expression analysis was carried out. The expression profile revealed that Class I TCP genes were expressed at lower levels in differentiating tissues and mature organs, while the *CgPCF2* gene was expressed at moderate levels in various tissues (**Figure 6A**). Among the Class II genes, three CIN genes (*CgCIN2*, *CgCIN5* and *CgCIN6*) exhibited high expression levels in flower organs (**Figure 6A**, **Supplementary Table 7**). In addition, the *CgCYC1* gene was only expressed in the stem, indicating that the Class II TCP genes function in plant development.

Flowers showed strong expression of class II genes such *CgCIN2*, *CgCIN5*, and *CgCIN6* (**Figure 6A**), indicating high expression and their potential significance in driving tissue differentiation. The gene expression of CIN homologs in *C. goeringii* was studied further in order to elucidate the precise functions of the Class II TCP genes by analyzing in the leaves, flowers, and stems using RT−qPCR (**Figure 6B** **;** **Supplementary Table 8**). The *CgCIN2* showed high expression in the sepals and lips, the *CgCIN5* showed high expression in the lips and columns, the *CgCIN6* showed extremely high expression in columns, of three examined CIN genes were barely detected in the stems and roots (**Figure 6B**). To understand the underlying functions of the TCP gene in *C. goeringii*, a functional study of these two classes will be a crucial next step.

## Discussion

As a traditional flowering plant, *C. goeringii* and their products have significantly contributed to the Chinese flower industry. However, the molecular biological mechanisms of orchid development have seldom been reported. TCP proteins are crucial for the development and growth of plants. Numerous plant species, including *A. thaliana* (Riechmann et al., 2000), *Hypoxis decumbens* (Madrigal et al., 2017), *P. equestris* (Lin et al., 2016), and *D. catenatum* (Zhang et al., 2021a), the TCP gene family have been discovered. However, the analysis of the TCP gene family in orchids was still insufficient. The TCP gene family of *C. goeringii* was identified using a variety of methods in this work, and the evolution of CgTCP proteins as well as their functional properties were examined.

Non-model plant gene family studies have greatly increased due to advances in whole-genome sequencing. This study identified 14 *CgTCPs* from *C. goeringii* and divided them into two main clades (**Table 1** and **Figure 1**). Compared with other orchids, the number of TCP genes in *C. goeringii* was less than that in *D. catenatum* (25) and *P. equestris* (23) (Lin et al., 2016; Zhang et al., 2021a). *C. goeringii* may have suffered gene loss during evolution. The three orchids have similar numbers of CIN genes but different numbers of PCF and CYC genes. There are 11 PCF genes in both *D. catenatum* and *P. equestris*, four PCF genes in *C. goeringii*, three CYC genes in *D. catenatum* and *P. equestris*, and only one CYC gene in *C. goeringii*. The different TCP gene numbers may be related to the life form. The physical and chemical properties of CgTCP proteins were similar to those of *D. catenatum* and *P. equestris*. All CgTCP proteins were predicted to be located in the nucleus, except for *CgCIN1*, which is also located in the chloroplast and may contribute to new functions. Furthermore, we performed the evolution of TCP genes in orchids and other land plants (**Supplementary Figure 1**). Previous study suggested TCP gene family may origin from the common ancestor of Phragmoplastophyta, and expanded through the whole-genome duplication during the evolution (Liu et al., 2019; Wang et al., 2022). Our results supported that lower plants have a smaller number of TCP genes and TCP family has undergone expansion in the course of plant evolution. We also investigated the evolution of orchid TCP genes and found that almost all subfamilies of TCP are present in orchids, and *D. catenatum* possessed more members compared with other orchids. Comparing with other monocots, orchids Class II-A genes were expanded, *PeCIN8* which located in Class II-A plays curial role in ovule development (Lin et al., 2016), the expanded genes may relate to orchid ovule unique development.

The conserved gene structure in the same clade of the TF gene family had been carried out in previous researches (Wang et al., 2021; Ke et al., 2021; Zhang et al., 2022; Chen et al., 2022). This study revealed that *C. goeringii* has an exon–intron structure similar to that of *Camellia sinensis* and *Prunus mume* (Zhou et al., 2016; Shang et al., 2022) (**Figure 4**). Almost all sequenced orchids have been reported to have long introns (Cai et al., 2015; Zhang et al., 2016; Zhang et al., 2017; Yuan et al., 2018; Ai et al., 2021; Sun et al., 2021; Zhang et al., 2021b; Li et al., 2022), but in *CgTCPs*, only four genes have long introns, and eight *CgTCPs* have no introns. The motif analysis of *C. goeringii*, *P. equestris* and *D. catenatum* indicated that class I, which contains motifs 1, 2, and 4, was conserved and that class II, which contains different motifs in different clades, was more variable (**Figure 4**). The conservatism of Class I genes may result in the crucial role in plants, which mostly exist in all phylostrata plants (**Supplementary Figure 1**). The *CgTCP* alignment was also analyzed in this study, and the results showed that the TCP domain and R domain were conserved in orchids (**Figure 2**).

Gene location in scaffolds and chromosomes and genomic comparison are closely related to the gene structure and function (Ren et al., 2000). The gene location analysis results showed that *CgTCPs* were scattered on eight chromosomes, and no gene tandem repeats were found in *C. goeringii* (**Figure 3A**). The genomic comparisons among *D. catenatum*, *P. equestris* and *C. goeringii* in chromosomes or scaffolds showed that their TCP genes were in an almost 1:1 correspondence, supporting that no duplication events occurred in *CgTCPs* (**Figure 3B**). In addition, *P. equestris* exhibited a more syntenic relationship between its scaffold or chromosome than *D. catenatum*, indicating that there were few chromosomal structure variations after the two species diverged.

Studies of promoter regions that regulate gene expression at the transcriptional level by *cis*-acting elements promote our basic understanding of gene regulation and expand the toolbox of available promoters (Hernandez-Garcia and Finer, 2014). A series of functional types of regulatory elements in the promoter regions have been identified in TCP genes, including the following categories: growth and development elements, stress-responsive elements and phytohormone-responsive elements (**Figure 5**). We identified a large proportion of light responsiveness elements in the promotor region, indicating that orchids TCP genes are regulated by the light signal to work in concert with plant growth and development (**Supplementary Table 4**). Furthermore, CgTCPs perform important functions regulated by phytohormones, which contain many elements responsive to MeJA, ABA, GA, and auxin. Based on previous studies, the TCP3, TCP14 and TCP15 genes regulate cell expansion and differentiation by auxin (Koyama et al., 2010; Ferrero et al., 2021), and CgTCPs may interact with auxin by a similar mechanism. In the inflorescence shoot apex and embryo root apical meristem, GA-induced DELLA protein degradation releases TCP genes to stimulate shoot elongation and seed germination (Nicolas and Cubas, 2016). In leaves, TCP-CIN genes interact with the CK receptor *HISTIDINE KINASE 4* (*AHK4*)/*CRE1* (*CYTOKININ RESPONSE 1*) and can promote the expression of the CK response genes *ARR7* and *ARR15* (Das Gupta et al., 2014). In seeds, the TCP14 gene could promote germination by regulating ABA signaling (Tatematsu et al., 2008). The *cis*-element analysis and GO analysis indicate that the TCP genes are mainly regulated by phytochromes and light and play an important role in different plant tissues of growth and development.

TCP transcription factors control key vegetation and reproductive developmental processes and participate in the growth patterns of meristems and organs (Nicolas & Cubas, 2016). Class II TCP genes contain genes mainly involved in lateral organ development (Crawford et al., 2004; Ori et al., 2007). In this study, we found and verified that *CgCIN2*, *CgCIN5* and *CgCIN6* were highly expressed in various flower components (sepal, petal, lip and column) and relatively highly expressed in leaves (**Figure 6** **;** **Supplementary Table 8**). Liu et al. (2017) found that the downregulation of five class II TCP genes may be related to the delayed flowering phenotype of *Arabidopsis* mutants. We speculate that *CgCIN2*, *CgCIN5* and *CgCIN6* may produce a marked effect on the regulation of florescence.

## Conclusion

TCP transcription factor proteins are known to play a crucial role in many aspects of plant growth and development. Here, we identified 14 TCP genes of *C. goeringii* and classified them into two clades by a phylogenetic analysis, with more members found in Class II. The sequence alignment, motif, gene structure and collinearity analyses indicated that *CgTCPs* were conserved and that no gene duplication events occurred in *C. goeringii*. The *cis*-element and GO analyses indicated that *CgTCPs* are regulated by light and various phytochromes. Furthermore, the expression pattern and RT−qPCR analyses suggested that *CgCIN2*, *CgCIN5* and *CgCIN6* may produce a marked effect on the regulation of florescence. Altogether, this study presents a comprehensive analysis of the structure and expression pattern of TCP genes in *C. goeringii*. These results provide a reference for further understanding how TCP genes participate in plant vegetative and reproductive growth and development and explain the plasticity of plant morphogenesis.

## Data availability statement

The datasets presented in this study can be found in Figshare, https://doi.org/10.6084/m9.figshare.21548541.

## Author contributions

The research was created and planned by SL and Z-JL. The data analysis was completed by D-KL and M-HL, who also prepared the paper. The analysis was carried out experimentally by CZ, XZ, QZ, and DZ. The plant materials were gathered by SK and YL. All authors contributed to the article and approved the submitted version.

## Supplementary material

Supplementary Data Sheet Sequences of *Cymbidium goeringii* TCP proteins.

Supplementary Figure 1 The phylogenetic tree of TCP genes based on 18 plant species.

Supplementary Figure 2 The entire map of the sequence alignment presentation of *Cymbidium goeringii* TCP proteins.

Supplementary Table 1 Prime used in RT−qPCR.

Supplementary Table 2 The TCP protein sequences of 18 plant species.

Supplementary Table 3 Detailed information of the *cis*-acting element prediction.

Supplementary Table 4 Statistics of the quantity and proportion of different types of *cis*-acting elements.

Supplementary Table 5 Statistics of *cis*-acting elements involved responsive functions.

Supplementary Table 6 GO annotation of the TCP genes of *C. goeringii*.

Supplementary Table 7 Expression pattern data in seven tissues of *C. goeringii*.

Supplementary Table 8 Statistical analysis using ANNOVA multiple comparisons for qPCR expression levels between reproductive organs and vegetable organs.
