# Supplementary figures and images for: Genome-wide analysis of the TCP gene family and their expression pattern in Cymbidium goeringii
Source: Front Plant Sci. 2022 Dec 8;13:1068969. doi: 10.3389/fpls.2022.1068969 (PMC9772009; doi:10.3389/fpls.2022.1068969)

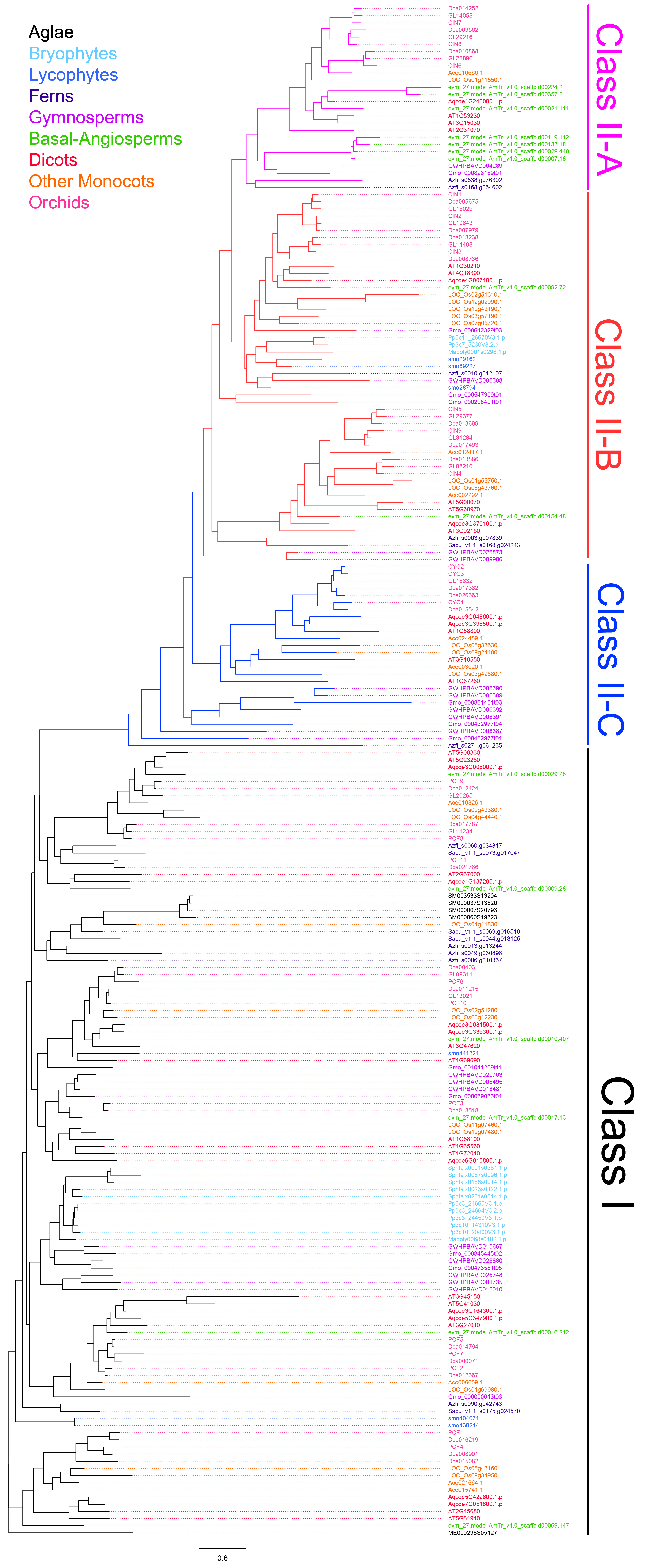

Supplement: Supplementary Figure 1 — The phylogenetic tree of TCP genes based on 18 plant species. [file Image_1.jpeg]

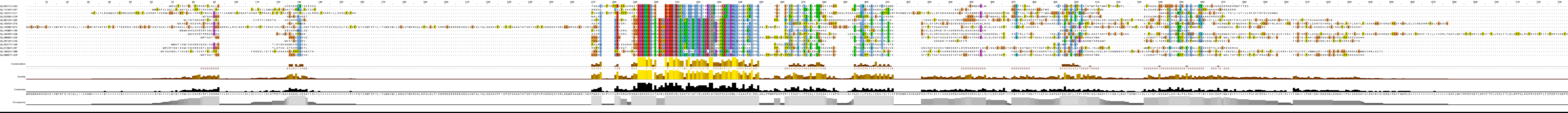

Supplement: Supplementary Figure 2 — The entire map of the sequence alignment presentation of Cymbidium goeringii TCP proteins. [file Image_2.png]
